# Voxel-Based Lesion–Symptom Mapping Localizes Residual Visual Function in Hemianopia

**DOI:** 10.1523/JNEUROSCI.1263-24.2024

**Published:** 2025-01-08

**Authors:** Hanna E. Willis, Junaid Hameed, Lucy Starling, Carina Kaltenbach, Margaret Jane Moore, Amirah Khan, Rachel Maxwell, Marco Tamietto, Sara Ajina, Holly Bridge

**Affiliations:** ^1^ Wellcome Centre for Integrative Neuroimaging, Nuffield Department of Clinical Neuroscience, University of Oxford, Oxford OX3 9DU, United Kingdom; ^2^ Department of Experimental Psychology, University of Oxford, Oxford OX2 6GG, United Kingdom; ^3^ Queensland Brain Institute, University of Queensland, Brisbane, Queensland 4072, Australia; ^4^ Department of Psychology, University of Torino, Torino 10124, Italy; ^5^ Department of Medical and Clinical Psychology, Center of Research on Psychology of Somatic Diseases, Tilburg University, Tilburg 5000, Netherlands; ^6^ Wellcome Centre for Human Neuroimaging, University College London, London WC1N 3AR, United Kingdom; ^7^ Department of Therapy and Rehabilitation, The National Hospital for Neurology and Neurosurgery, London WC1N 3BG, United Kingdom

**Keywords:** blindsight, hemianopia, motion, primary visual cortex (V1), residual vision, voxel-based lesion–symptom mapping (VLSM)

## Abstract

Damage to the primary visual cortex (V1) results in visual field deficits on the contralateral side of the world corresponding to the damaged region. Patients with such loss nonetheless show varying residual vision within this apparently blind region, with the neural mechanisms underlying this ability obscured by small study populations. We identified lesions on structural scans from 39 patients (12 female) with hemianopia and occipital lobe damage. We estimated the proportion of damage throughout the visual cortex and correlated this with performance in up to three different tests of residual vision in the blind field. We then performed voxel-based lesion–symptom mapping (VLSM) to determine the precise neural regions associated with residual vision. V1 damage did not show a relationship with residual vision measured with any task, although the extent of damage to V4 and hMT+/V5 both correlated with the ability to detect contrast-modulated stimuli. Indeed, damage to hMT+/V5 severely impacted performance across all three tasks, particularly motion detection. Although hMT+/V5 appeared necessary for residual vision, some patients with intact hMT+/V5 had no residual vision, indicating this area alone was not sufficient. VLSM revealed that damage to the optic radiation laterally was most highly associated with poor residual vision. Thus, VLSM indicates that hMT+/V5 and the optic radiation specifically are critical for residual vision in hemianopia.

## Significance Statement

Visual loss is extremely common after acquired brain injury but is notoriously difficult to treat. Residual vision, or “blindsight,” represents a potential target for visual retraining and plasticity. In the largest single-method study in blindsight, we apply the technique of voxel-based lesion–symptom mapping for the first time. We show that the extrastriate cortex is important for residual vision, but its involvement in lesions cannot explain all behavioral results. Instead, a region of optic radiation close to the visual motion area and lateral occipital cortex is most highly associated with residual vision in hemianopia. This represents a potential target for hemianopia recovery training and may help us understand why some patients benefit from extensive visual retraining while others do not.

## Introduction

Over the past century, the study of patients who have damage to specific regions of the brain has been fundamental for investigating the localization of function. Within the visual system, it has led to an increase in understanding about specialization of function in restricted areas of the occipital lobe, for example, the role of the lateral occipital regions in object recognition by studying visual agnosia (James et al., 2003; Bridge et al., 2013) and the role of the ventral regions in face processing by studying prosopagnosia (Barton, 2008). However, when the damage includes the primary visual cortex (V1), the most obvious deficit is a loss of visual field, whether full hemianopia, quadrantanopia, or a smaller scotoma. In this case, despite an apparent loss of vision as measured by clinical perimetry tests, some patients retain the ability to detect or even discriminate visual stimuli within the blind field (Stoerig, 2006; Sanchez-Lopez et al., 2020). This is known as “blindsight” (Weiskrantz et al., 1974; Weiskrantz, 1986; Stoerig and Cowey, 1997). Traditionally “blindsight” refers to residual abilities without awareness [also known as Type 1 blindsight (Weiskrantz et al., 1974)]. Here, we use the term residual vision to refer to evidence of above-chance responses to visual stimuli presented within the affected hemifield, regardless of whether or not participants were aware of these visual stimuli (Mazzi et al., 2016).

The presence of residual vision in people with hemianopia depends on a wide range of factors, including the size and location of the lesion. Functional MRI studies have implicated a number of extrastriate areas in visual processing after damage to V1. A meta-analysis of fMRI studies reported significant activity in subcortical and extrastriate areas in the dorsal and ventral stream, consistent with a network-based organization in the functional neuroanatomy of blindsight (Celeghin et al., 2019). Particular attention has been devoted to characterizing the role of motion area hMT+ and its subcortical efferents. Recently neurochemistry in hMT+ has been found to be related to residual vision (Willis et al., 2023). Moreover, both the structural integrity of the pathway between the lateral geniculate nucleus (LGN) and hMT+ (Ajina et al., 2015b) and functional connectivity between these two structures (Ajina and Bridge, 2018) appear to relate to residual visual ability. Visual rehabilitation using motion discrimination led to an improvement in the white matter microstructure of this tract (Willis et al., 2024b). Baseline activity in hMT+ (Ajina et al., 2021) and perilesional V1 (Barbot et al., 2021) has also been shown to relate to the extent of improvement following extensive visual training. Similarly, resting state connectivity between the precuneus and posterior occipital cortex at baseline is related to the extent of improvement on a training paradigm that required discrimination either of the location of a static point or direction of an optic flow pattern (Halbertsma et al., 2020). However, hMT+/V5 may not be important for blindsight involving static, colored stimuli (Morland et al., 1999; Alexander and Cowey, 2010), which have been shown to elicit activation in the ventral stream of patients including the lateral occipital cortex and V4/V8 (Goebel et al., 2001). Finally, the technique of lesion network mapping has been used to translate reconstructed 2D lesions of blindsight patients taken from published studies to resting state data from healthy participants (Kletenik et al., 2022). Connectivity to the ipsilesional medial pulvinar was the only significant predictor of blindsight occurrence; however, the blindsight tests varied widely, and this indirect technique would be less sensitive to the differences in residual LGN connectivity due to its involvement in the primary visual pathway and hemianopia.

Since the majority of studies performed on participants with blindsight have small group sizes (usually smaller than *n* = 10), it is difficult to establish the brain regions critical for residual visual function. Here, we collate structural MRI data and corresponding behavioral measures of residual visual function from a comparatively large sample of participants with visual field deficits (*n* = 39), collected over 10 years. All had been assessed for residual vision using the same moving contrast detection task, enabling us to perform voxel-based lesion–symptom mapping (VLSM) for the first time. A subset of participants also undertook behavioral tasks to detect moving dots and face/place stimuli, which provided an opportunity to determine whether the specific location of damage affects residual vision for different types of stimuli.

We hypothesized that (1) the extent of occipital damage was likely to correlate with performance on the contrast detection task and (2) damage that extended into the dorsal or ventral extrastriate regions would specifically affect performance on the motion or face/place tasks, respectively.

## Materials and Methods

### Participants

Thirty-nine participants with damage to V1 resulting in homonymous visual field deficits were included in this analysis. All participants were recruited to one of three studies investigating hemianopia and residual vision undertaken between 2013 and 2022. In each case, participants were aged 18–80 and had suffered damage to the brain resulting in homonymous visual field deficits (including, hemianopia, quadrantanopia, and scotoma). The main exclusion criterion was the presence of any other neurological or noncorrectable visual deficit. Each participant provided informed consent, and all experiments were conducted in accordance with the ethical guidelines of the Declaration of Helsinki.

Seventeen participants were scanned between 2013 and 2016, from whom diffusion-related and fMRI data have already been published (Ajina et al., 2015a,b,c; Ajina and Bridge, 2018, 2019). Ethical approval was provided by the Oxfordshire Research Ethics Committee (Ref B08/H0605/156). Of the 17 participants (five female), 15 had sustained posterior circulation stroke, and 2 had undergone benign tumor resection.

Nine participants (four female) were scanned between 2017 and 2018 in a study investigating population receptive field mapping in hemianopia. Six of these participants had suffered ischemic stroke resulting in visual field loss, and the other three had hemorrhagic strokes. The study was approved by the University of Oxford Interdivisional Research Ethics Committee (IDREC; R59810/RE001).

Thirteen participants (three female) were recruited as part of a visual rehabilitation study (Willis et al., 2024b). All acquired visual field deficits following stroke (six ischemic, four hemorrhagic, three unknown cause). Other data from this study have been published (Willis et al., 2023, 2024a), while data used here are from the baseline scan session, acquired between 2021 and 2022. The study was approved by the University of Oxford IDREC (R60132/RE001).

The average age (±SD) of all participants at the time of participation was 51 ± 14.9 years. Average time after pathology onset was 40 months (range, 6–297 months). Data from all participants were combined for the analyses as psychophysical testing was identical and variability in scanning sequences is unlikely to greatly affect lesion identification in structural images. Participant characteristics are provided in Table 1.

**Table 1.** Participant characteristics

Participant	Sex	Age band	Lesion side	Blindsight	Cause	Months since lesion
R001	M	50–54	R	Y	PCA stroke	45
R002	F	25–29	L	N	PCA stroke	14
R004	F	45–49	R	N	Hemorrhagic	18
R005	M	60–64	R	N	Hemorrhagic	6
R006	M	20–24	R	N	Ischemic	13
R007	M	45–49	R	Y	Ischemic	7
R008	M	40–44	R	N	PCA stroke	9
R009	M	35–39	R	Y	PCA stroke	25
R010	M	60–64	L	N	Ischemic	26
R011	M	60–64	R	N	PCA stroke	7
R012	M	70–74	R	N	PCA stroke	31
R013	M	70–74	L	N	Hemorrhagic	26
R014	F	35–39	L	Y	Ischemic	30
R015	F	35–39	L	Y	Ischemic	8
R017	M	65–69	L	N	Ischemic	6
R018	M	35–39	L	N	Ischemic	26
R019	F	40–44	R	N	Ischemic	31
R020	F	45–49	R	Y	PCA stroke	297
R022	M	45–49	L	N	Ischemic	14
R023	M	65–69	L	Y	PCA stroke	49
R024	M	60–64	R	Y	Ischemic	13
P012	F	60–64	L	N	PCA stroke	28
S202	M	75–79	L	N	Tumor resection	252
S203	M	75–79	L	Y	Ischemic	20
S204	F	65–69	R	Y	Hemorrhagic	18
S205	F	40–44	L	Y	Ischemic	19
S206	F	35–39	R	Y	Tumor resection	54
S208	M	65–69	R	N	Ischemic	24
S209	M	55–59	L	Y	Ischemic	18
S210	M	50–54	L	N	Ischemic (iatrogenic)	84
S211	M	65–69	R	Y	Ischemic	6
S214	M	35–39	L	Y	Ischemic	12
S215	M	70–74	L	Y	Ischemic	19
S216	M	55–59	L	Y	Ischemic	96
S217	M	30–34	L	Y	Ischemic	156
S218	M	70–74	R	N	Hemorrhagic	15
S219	M	55–59	R	N	Ischemic	36
S220	F	35–39	L	Y	Ischemic	7
S401	F	40–45	L	Y	Ischemic	12

Posterior cerebral artery (PCA) stroke is used where it is not known whether the stroke was ischemic or hemorrhagic.

### Psychophysical testing

**Figure 1.**
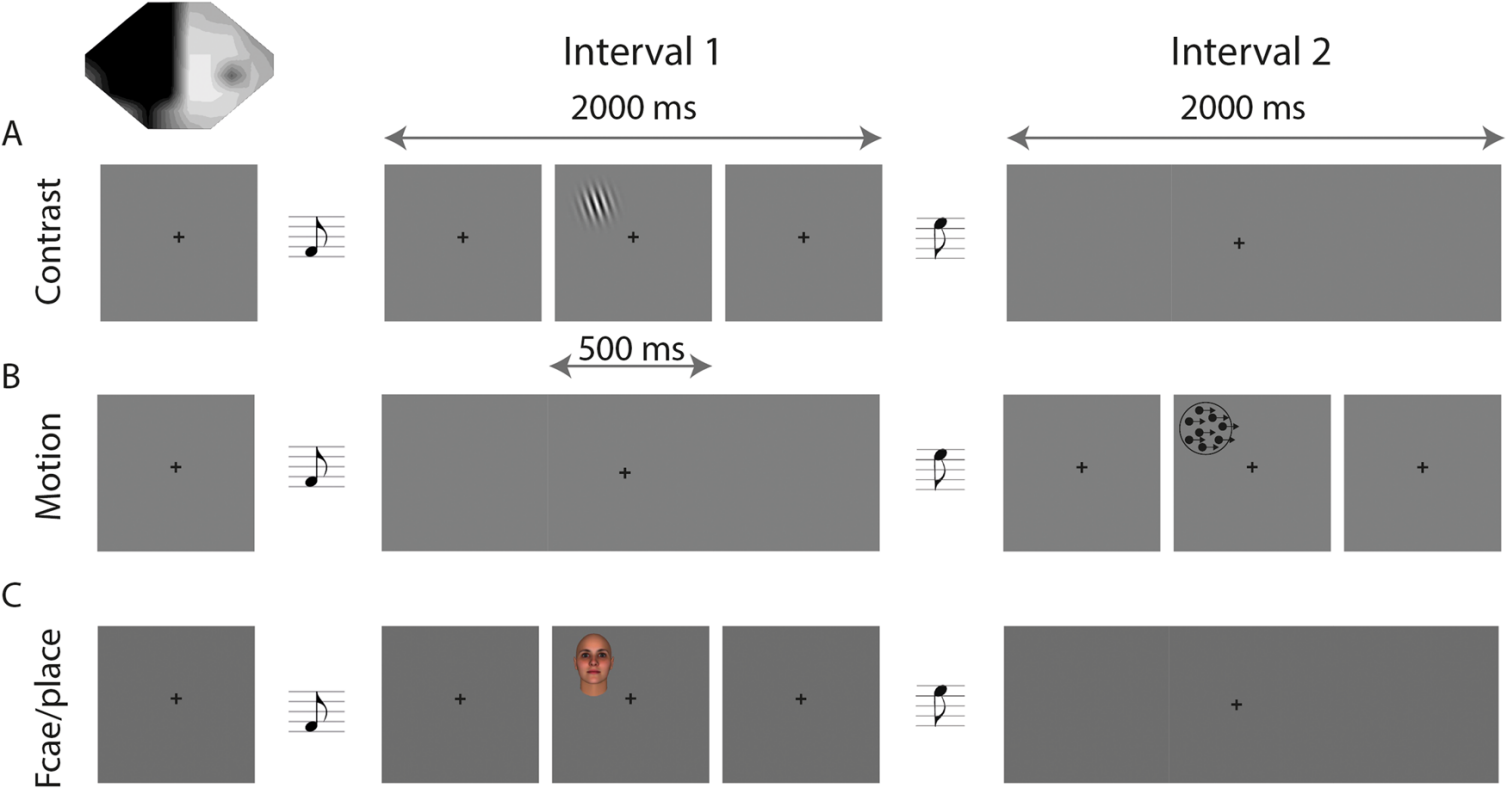
Behavioral testing protocol. In each case, participants had to indicate whether they saw the stimulus in Interval 1 or Interval 2, with the two intervals indicated by a different frequency tone. Note that both intervals are 2,000 ms long and identical, except that the stimulus appears for 500 ms in one of the intervals. In the contrast task (***A***), the stimulus consisted of a drifting Gabor patch of varying contrasts. In the motion task (***B***), the stimulus was a patch of dots that moved coherently at different speeds, and the face and place task (***C***) involved presentation of either a face or a place. The visual field in the top left corner shows an example of the location of the visual field deficit for one participant.

The loss of visual field was defined using standard clinical Humphrey perimetry. Three two-alternative forced-choice (2AFC) psychophysical detection tasks were used to measure residual vision in the perimetrically defined blind fields: (1) drifting contrast detection likely to activate early visual areas, (2) moving dot detection targeting the motion-selective dorsal visual regions, and (3) static face and place detection targeting the ventral visual stream. In each 2AFC detection task, participants were required to indicate whether the stimulus appeared in the first or second time interval. The stimulus diameter was either 5° or 8°, placed fully within the perimetry-defined region of field loss on a uniform gray background with a luminance of 50 cd/m^−2^. The mean luminance of Gabor patches and face/place stimuli also matched the midgray background. The onset of each interval was indicated by a 500 ms auditory tone, 300 Hz marking the onset of the first interval and 1,200 Hz for the second. Stimuli appeared for 500 ms with jittered onset within a range of 500–1,500 ms while the participant fixated on a central black cross. Figure 1 shows a schematic representation of the tasks. All participants completed the contrast detection task, while the subgroups completed the other two detection tasks.

#### Contrast detection task

For the contrast task (number of participants, 39), a drifting achromatic Gabor patch (temporal frequency, 10 Hz; spatial frequency, 1.3 cycles/°; stimulus diameter, 5°; duration, 500 ms) of varying luminance contrasts (1, 5, 10, 50, 100%; 10 trials of each) was presented. Only performance on the “high” contrasts (50 and 100%) was used to quantify residual vision, as most patients only show above-chance detection for high-contrast stimuli (Ajina et al., 2015a,c, 2021; Ajina and Bridge, 2019; Kletenik et al., 2022). Participants were categorized as “at chance,” if their result was nonsignificant at 5% using a cumulative binomial distribution. The same method was employed for each of the three detection tasks. For the motion and face/place task, we took an average detection performance across all stimuli. For correlation analyses, any psychophysical results <50% that were not statistically different from chance were assigned 50%. This was the case for 6/39 participants in the contrast detection task (lowest value, 35%); 7/24 for the motion task (lowest value, 45%), and 2/19 for the face/place task (lowest value, 42.2%). This was a conservative approach to prevent spurious correlations.

#### Motion detection task

For the motion task (number of participants, 24), a patch of moving black dots (luminance, 0.5 cd/m^−2^; stimulus diameter, 5°; dot size, 0.075°; lifetime, 12 frames; density, 8 dots/deg^2^) of variable speed (4, 8, 20, and 32°/s) was presented. Due to a lack of consensus on optimal dot size and density in blindsight (Beckers and Zeki, 1995; Weiskrantz et al., 1995; Azzopardi and Cowey, 1998; Zeki and Ffytche, 1998), stimulus parameters were based on classic descriptions of coherent motion in human MT (Rees et al., 2000; Braddick et al., 2001). Since the experiment was a detection task, performance was collapsed across all speeds to increase the number of trials used in the metric.

#### Faces and place detection task

For the face and place detection task (number of participants, 19), visual stimuli consisted of two sets of eight unique images, one depicting human faces and another of simple outdoor scenes representing the category of “places.” Face stimuli were colored images of emotionally neutral, expressionless faces taken from a set of randomly generated images using the Facegen Modeller program (http://facegen.com) version 3.1. The exact procedures including model validation are described by Oosterhof and Todorov (2008). Eight different face identities were chosen, each covering an ellipsoid area subtending either 7.25 or 5° in height. Simple scene images were taken from the revised Snodgrass and Vanderwart's object databank, with color and texture previously added to the original images for improved recognition (Rossion and Pourtois, 2004). Images were not naturalistic and varied in their contour outline, with each image superimposed directly on the gray background. Eight images were selected to represent outdoor places, subtending between 6–7.25° and 4.1–5° visual angle. The mean area was similar across all stimuli, with “place” stimuli containing 86,125 pixels (±15,568 SD) and “face” stimuli containing 91,266 pixels (±3,475 SD), compared with Gabor and dot apertures containing 92,940 pixels. All images were also scrambled for use as controls, by applying a 2D Fourier transform. Scrambled images subtended 7.25° for large stimuli and 5° for the smaller set. Scrambled places and faces were combined as there was no difference between performance on the two tasks. Since there was no significant difference between the detection of faces and places (Wilcoxon sum of ranks, 38; *p* = 0.3), performance was collapsed across these two categories.

### Imaging acquisition

Seventeen participants were scanned between 2013 and 2016 on a 3 T Siemens Verio scanner at the Wellcome Centre for Integrative Neuroimaging, University of Oxford. A structural scan was acquired for each participant. This was a 1.04 × 1.04 × 1 mm^3^ whole-head T1-weighted MPRAGE anatomical image (TE, 4.68 ms; TR, 2,040 ms; field of view, 200 mm × 200 mm; flip angle, 8°).

Twenty-two participants were scanned between 2017 and 2021 on a 3 T Siemens Prisma scanner in the same location, and the same scan sequence was used for both groups: 1 mm isotropic whole-head T1-weighted MPRAGE anatomical image (TE, 3.97 ms; TR, 1,900 ms; field of view, 192 mm × 192 mm; flip angle, 8°).

### Imaging analysis

#### Lesion mask definition

The lesion size and location were initially quantified for all participants. Given the variability in the visual appearance of lesions, manual delineation was used to identify the lesion in each participant. Three or four researchers independently identified lesions on each participant's native space scan. Researchers were instructed to define the lesion as the area of damaged tissue (including damaged white matter). Where the lesion was continuous with the lateral ventricle, researchers were instructed to define the lesion assuming the normal size of the ventricles was approximately comparable across the two hemispheres.

**Figure 2.**
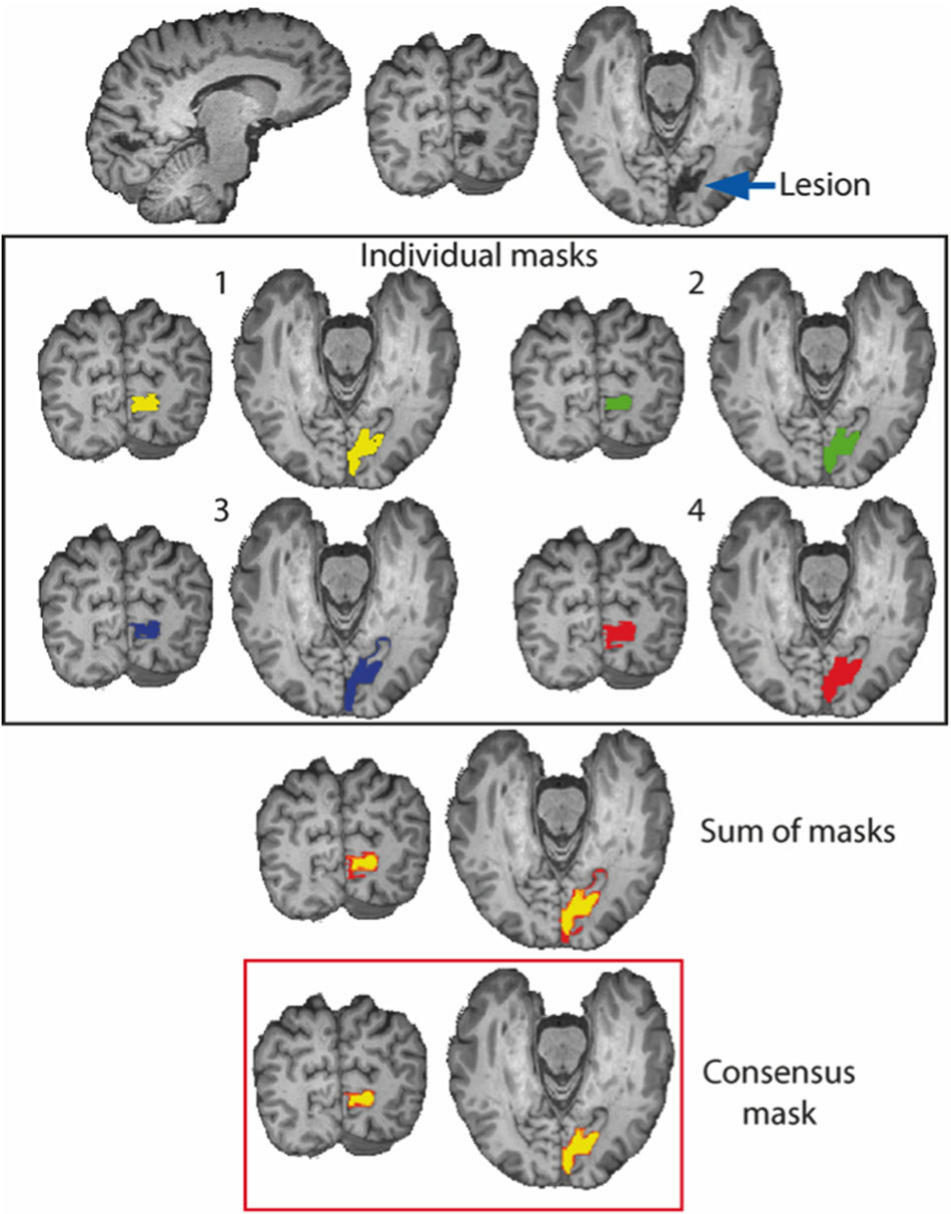
Creation of the lesion mask used for all analyses. Four individuals independently identified the lesion. The four masks were then summed and thresholded so that only voxels labeled by at least two individuals were included in the final lesion mask “consensus mask.”

Lesion definitions across the four individual lesion masks were summed and then thresholded at a value of 1.9 so that only voxels identified by at least two researchers were included in the final consensus lesion mask. Figure 2 shows the pipeline with examples of the lesions and mask.

#### Regions of interest

Visual areas V1 and V4 and hMT+/V5 were extracted from the Jülich atlas, which provides histologically defined regions, and the occipital cortex mask was extracted from the MNI structural atlas, both implemented in FSL (fmrib.ox.ac.uk/fsl). V1 and V4 can be mapped using retinotopic mapping and correspond well to the relevant probabilistic map used here. In contrast, mapping around the probabilistic map of hMT+/V5 indicates that the area likely includes both retinotopic areas TO-1 and TO-2 (Amano et al., 2009; Weiner and Grill-Spector, 2011).

Structural images were nonlinearly registered to MNI space using FLIRT (Jenkinson et al., 2002) and FNIRT, and the inverse warp was used to transform the regions of interest into the individual participant structural space. The proportion of the occipital cortex and each visual area that overlapped with the consensus lesion mask was then calculated as a measure of the extent of damage using the *fslmaths* tool.

**Figure 3.**
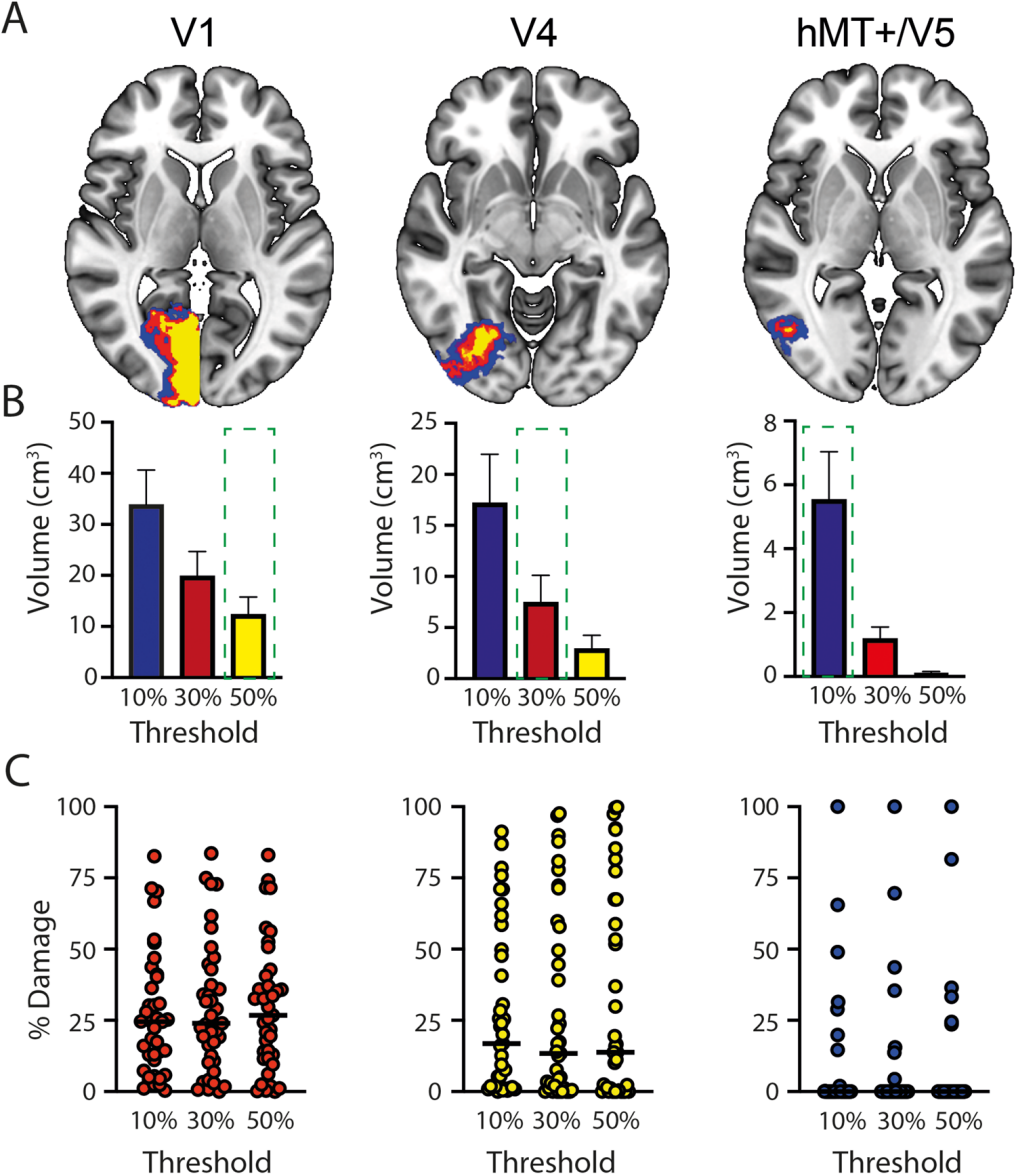
The effect of visual area thresholding on the percentage of damage in each area. ***A***, The size of the visual area mask extracted from the Jülich histological atlas implemented in FSLeyes varies considerably depending on the threshold used. ***B***, The volume of each visual area across participants at the different threshold values. Note that the relationship between visual area volume and threshold differs across the visual areas depending on the intersubject variability in position. V1 is the most consistent and hMT+/V5 the least. The dotted green box shows the selected threshold for each case. ***C***, The percentage of V1, V4, and hMT+/V5 damaged in each participant at each threshold. The median values show very little variability across the different thresholds.

As the Jülich atlas masks are probabilistic, it is important to determine the most appropriate threshold to use. In each case, the threshold reflects the overlap of the areas defined histologically in 10 postmortem brains, such that a threshold of 50% indicates that any voxels within the mask were present in at least 6 out of 10 participants. Based on prior experience, neuroanatomy and compatibility with other atlases (Glasser et al., 2016) thresholds of 10% (hMT+/V5), 30% (V4), and 50% (V1) were used. However, to ensure that the results were not biased by the specific threshold, the Jülich masks were thresholded at each of 10, 30, and 50% for V1, V4, and hMT+/V5. The effect of thresholding reflects the amount of variability in the location of the structure, where V1 is very consistent, and hMT+/V5 is the most variable across brains. Thresholding did not make a significant difference to the analysis since the proportion of overlap did not differ according to the threshold in any of the visual areas (V1, Friedman statistic = 6.6; *p* = 0.04; V4, Friedman statistic = 0.7; *p* = 0.70; hMT+/V5, Friedman statistic = 4.2; *p* = 0.12; Bonferroni-corrected *p* = 0.016). Figure 3 shows the effects of thresholding on the size of the brain region in standard space (A), the mean area volume across all participants (B), and the proportion of the visual area damaged by the lesion (C).

#### Voxel-based lesion–symptom mapping (VLSM)

VLSM analysis was conducted to identify regions associated with residual visual performance on the contrast detection task using LESYMAP (https://github.com/dorianps/LESYMAP). One participant was excluded from this analysis due to the damage being bilateral. All the brains with damage to the left hemisphere (*n* = 21) were flipped using the FSL tool *fslswapdim* along the *x*-axis to ensure that the lesions were conventionally assigned to the same hemisphere (right) for all participants. An overlap map was then made by combining the summed, binarized lesion masks from the remaining 38 participants (Fig. 2).

The relationship between lesion location and demeaned drifting contrast detection performance was evaluated using voxel-level lesion mapping analyses. These analyses aim to identify voxels that, when damaged, are associated with impaired performance on the drifting contrast detection task (Moore et al., 2023). Specifically, each lesioned voxel is assigned a score of 1, and each spared voxel is assigned a score of 0. These scores are then weighted to control for stroke severity using direct total lesion volume control (Zhang et al., 2014). At each voxel, a regression analysis is conducted to measure the degree to which the weighted voxel damage score predicts behavior. In cases where these analyses survive 5% false detection rate (FDR) corrections for multiple comparisons, the voxel is significantly associated with task performance. All voxels impacted in at least three participants were considered in these analyses.

### Statistical analysis

Correlations were performed in GraphPad Prism using Spearman rank correlations to determine whether residual vision on each task was related to damage to specific visual areas. To determine whether a straight line was the optimal fit to the data, a least-squares regression of residual vision against the proportion of damage was performed using both a second-order polynomial and a straight line. A sum-of-squares *F*-test was used to determine the better fit to the data. Nonparametric tests were used given that all psychophysical measures and the lesion quantification were percentage correct measures, and therefore non-normally distributed.

### Data availability

All anonymized data are available on request from the authors following publication.

## Results

**Figure 4.**
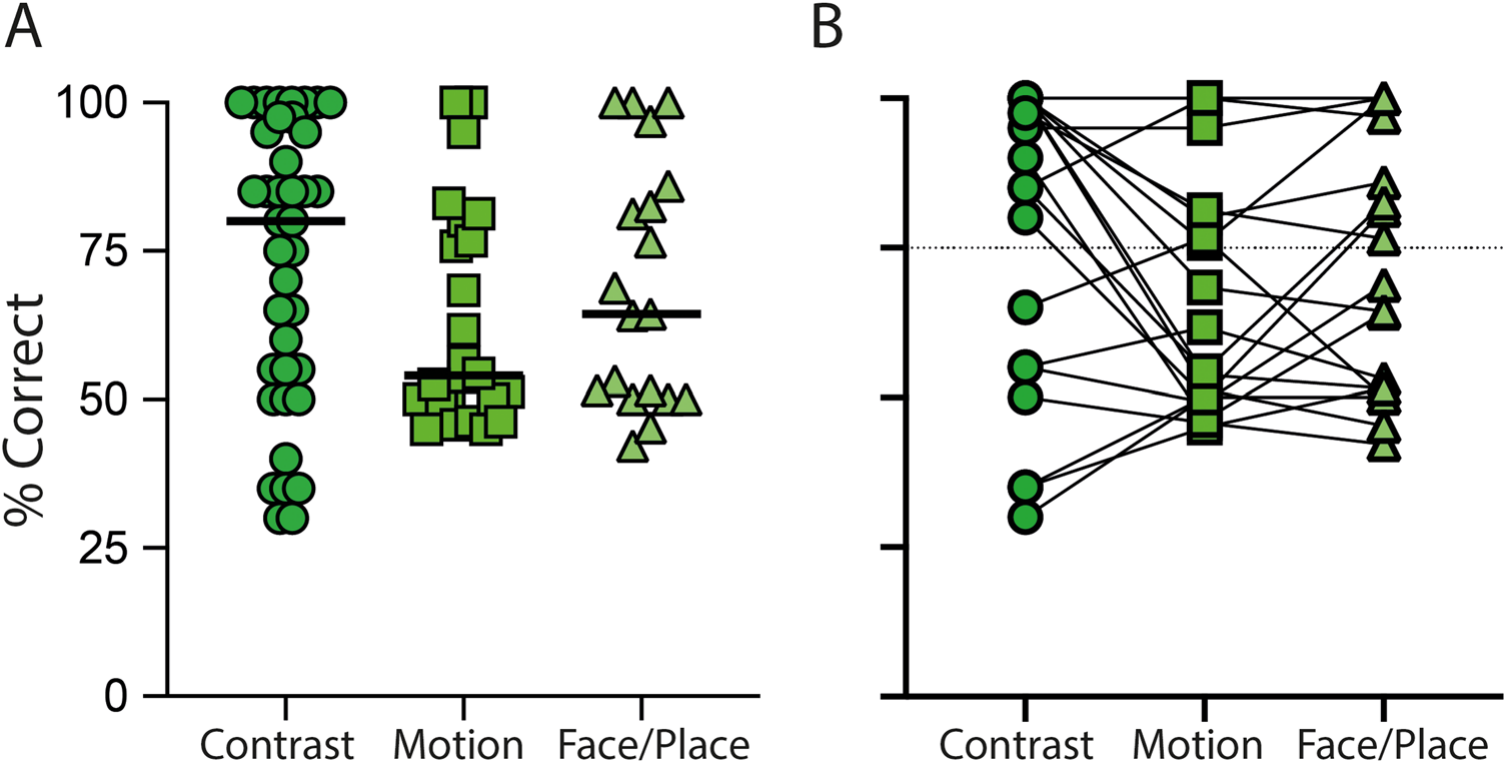
Behavioral performance across the three tasks. ***A***, The performance of all participants who participated in any of the experiments: contrast detection, motion detection, and detection of face or place stimuli. ***B***, The participants who completed all three tasks such that performance can be compared across tasks.

Participants showed a range of residual vision on the three behavioral detection tasks, from those able to detect stimuli 100% of the time to those performing at chance. Figure 4*A* illustrates the performance of each of the participants across the tasks. Performance was 85% [65%; 95%; median (lower and upper 95% CI)] on the contrast task, 54% (50%; 77%) on the motion detection task, and 64% (50%; 86%) on the faces and place task. In each case, 50% was chance performance, and values ≤25% and ≥75% were significantly different from chance using a binomial distribution. Figure 4*B* allows the comparison of performance for the 19 participants who performed all three tasks. While a Friedman test showed no significant difference across tasks (Friedman = 1.4; *p* = 0.49), it is clear that for some participants, the motion task performance was considerably reduced, although this was not the case for all. For later correlation analyses, chance values <50% were assigned to 50% to prevent artifactual correlations.

**Figure 5.**
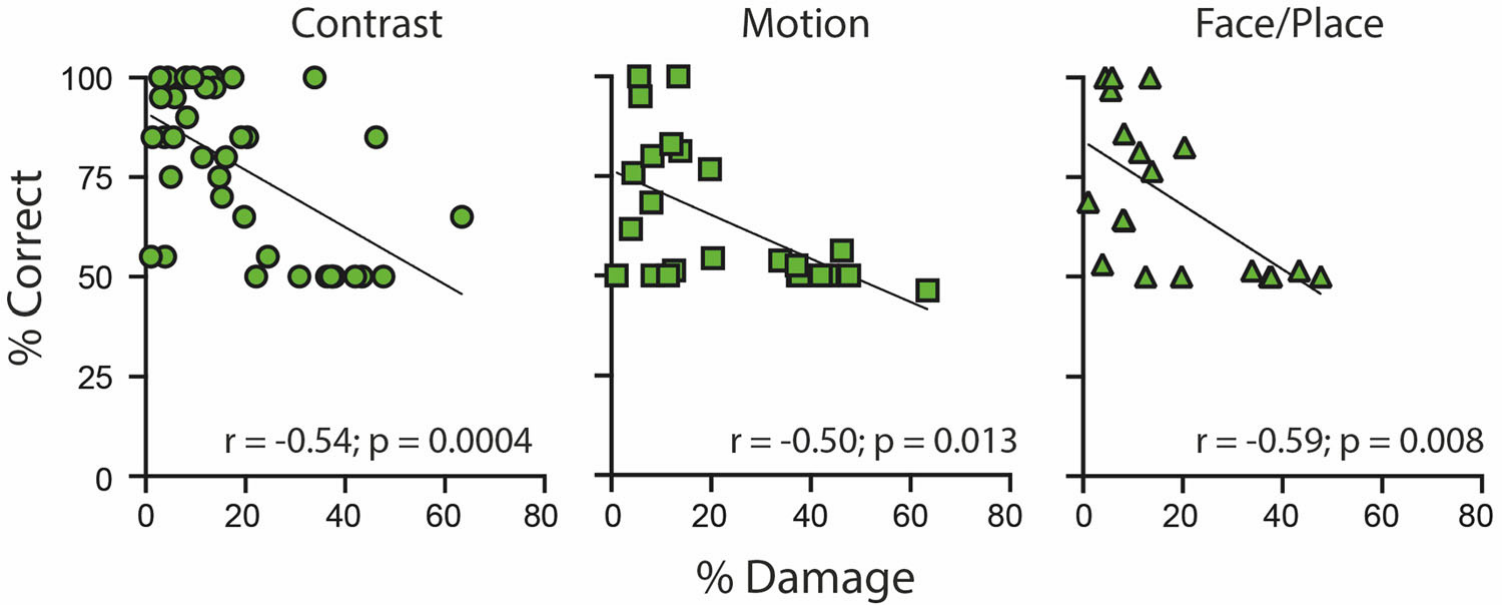
Correlation between the percent damage to the affected side of the occipital cortex and performance on the three psychophysical tasks. The significance level was set at *p* < 0.0167 to account for the multiple tasks and performance on all was significantly correlated with the amount of damage.

To determine the effect of overall damage to the occipital cortex on visual performance, the initial analysis was a basic correlation of the percentage of occipital damage and psychophysical performance on the three tasks. The percentage varied from 1 to 63% of the affected occipital lobe. Figure 5 shows the correlations for each task, all of which reach significance (*p* < 0.0167; Bonferroni corrected for three tasks). In cases where damage exceeded 20% of the occipital lobe, patients no longer showed any residual vision in the motion and face/place tasks. The contrast task, however, indicates that a couple of participants can still perform above chance even with 30–40% damage to the affected occipital cortex. It is also the case that a reasonable number of patients with relatively little occipital damage (<10%) show no evidence of residual vision, suggesting that lesion size alone cannot be the only factor.

**Figure 6.**
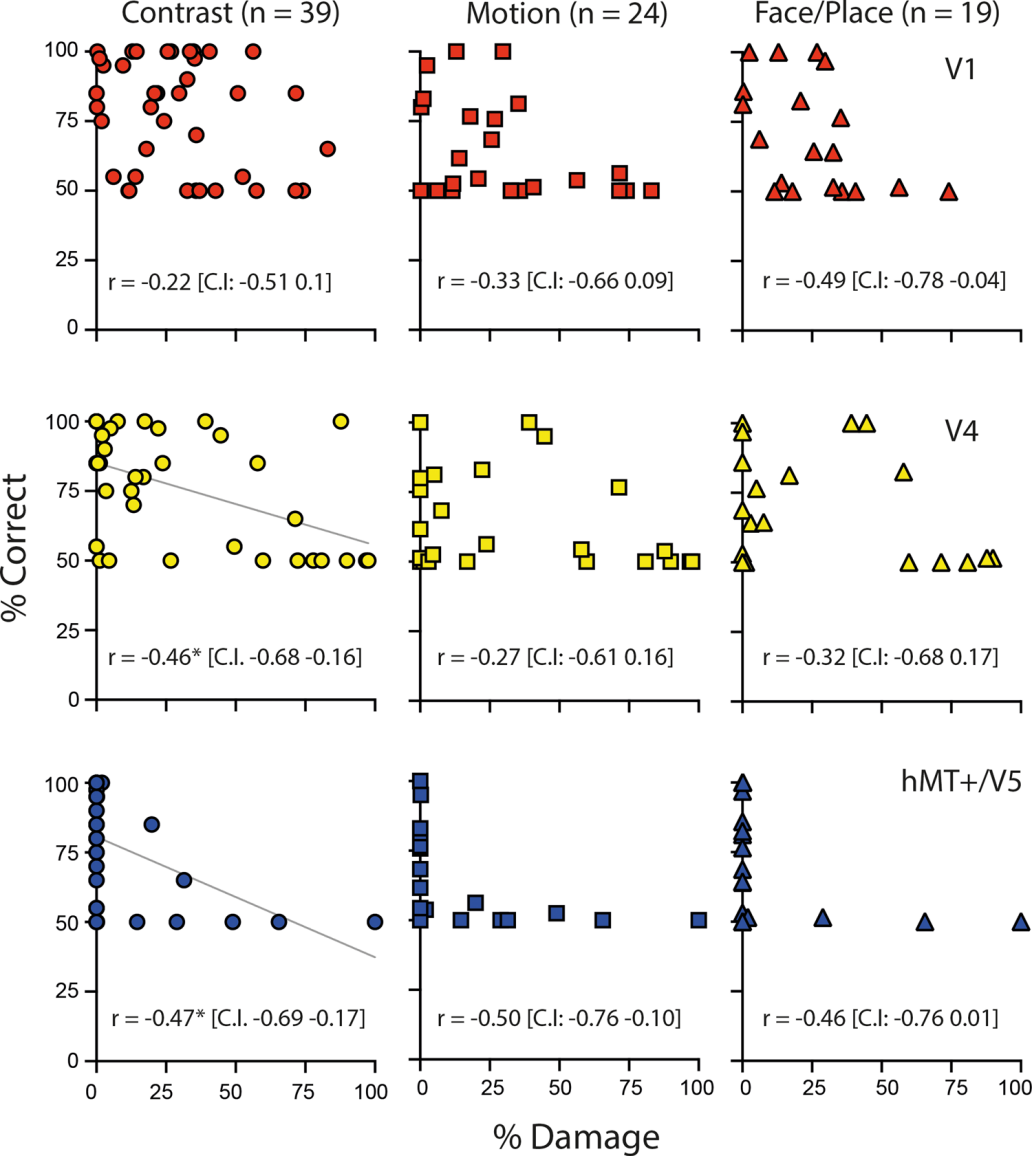
Relationship between brain region damage and behavioral tasks. Correlation between the percentage of damage to each visual area (V1, V2, V4, and hMT+/V5) and the percent correct for each of the three behavioral tasks: contrast (circles), motion (squares), face/place (triangles). * indicates correlations significant at *p* < 0.05 (Bonferroni corrected for 9 comparisons; *p* < 0.006). In each case, % correct below 50% has been assigned the value of 50% to prevent spurious correlations. The best-fit lines are indicated for significant relationships.

The relationship between residual visual performance and damage to each individual visual area was determined by correlating these measures (Fig. 6). There was no relationship between performance and extent of V1 damage for any of the tasks. This is perhaps not surprising since the task was performed within a region of the visual field affected by the damage.

While our hypothesis was that damage to V4 would have a stronger relationship with face/place stimuli compared with Gabor or motion stimuli, it was only the ability to detect contrast (Gabor) stimuli that correlated with the extent of damage in V4. There were, nonetheless, a number of participants with up to 50% of damage to V4 who were still able to detect stimuli across all three tasks.

**Figure 7.**
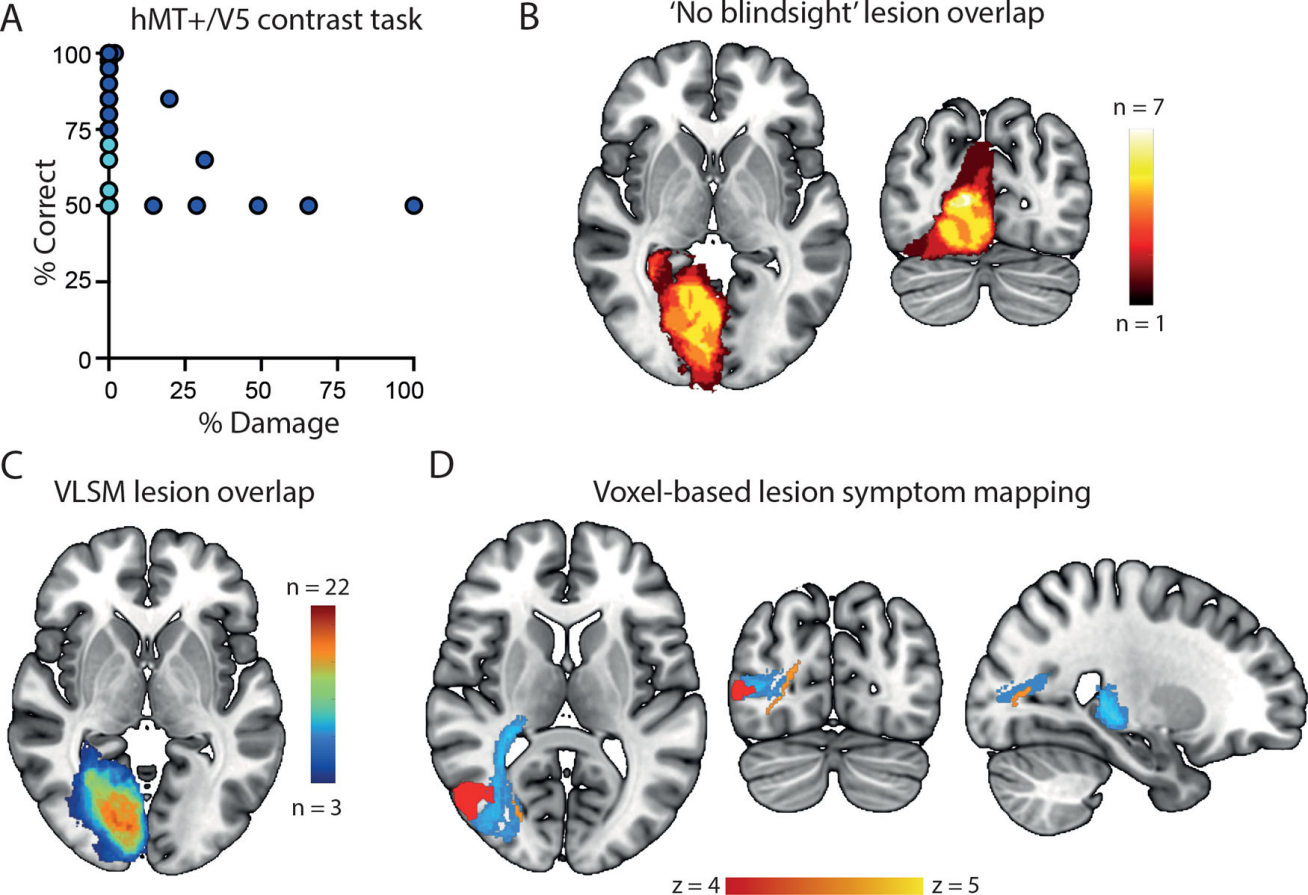
Voxel-based lesion–symptom mapping. ***A***, The correlation between the % damage to hMT+/V5 and performance on the motion detection task. The data in light blue are from patients who perform at chance despite not having any damage to hMT+/V5. Note that there are seven participants in this category but the points are overlapping. ***B***, The lesion overlay of the participants without damage to hMT+/V5 performing at chance. The lesions are relatively large, affecting much of V1. The voxel color represents the number of overlapping lesions. ***C***, The overlap of all 39 brains included in the VLSM, thresholded where lesions from at least three patients overlap. ***D***, The significant voxel cluster (*n* = 188; FDR-corrected) in the optic radiation yielded by univariate lesion–symptom mapping is shown in orange–yellow with area hMT+/V5 in red. The light blue tract is taken from a previous diffusion study tracking between LGN and hMT+/V5 (Willis et al., 2024a). While the region identified in the VLSM is not in the center of this tract, there is some overlap.

The extent of damage to hMT+/V5 also showed a significant correlation with performance on the contrast task, although the correlation values were similar across the three tasks. Indeed, participants with “any” damage to hMT+/V5 (*n* = 8; average damage, 39%; range, 2–100%) performed at chance on motion and face/place detection tasks. Therefore, damage to hMT+/V5 appears to significantly impact residual visual ability. However, we also found that some participants with no damage to hMT+/V5 also performed around chance (50%) on the tasks. Figure 7*A* highlights in light blue the participants without any damage to hMT+/V5 who performed at chance in the contrast detection task. There were seven participants who met this criterion, and the overlap of their lesions is shown in Figure 7*B*. To explore whether damage to other regions of the visual system underlies this lack of residual vision, we performed a whole-brain voxel-based lesion–symptom mapping analysis (VLSM). Figure 7*C* shows the lesion overlay from the 39 participants with a rainbow map reflecting the number of participants with damage in any given voxel. Overall, a volume of 14.7 cm^3^ was damaged in at least three patients and was therefore included in the analyses. The maximum overlap was 22 participants, located in V1 around the calcarine sulcus. Figure 7*D* shows, using univariate VLSM analysis, the cluster of voxels which, when damaged, are associated with reduced residual visual function in the contrast detection task. The voxels are located within the white matter of the optic radiation and have a volume just over 1,300 mm^3^ (1,328 voxels). The Jülich white matter tractography atlas (Hua et al., 2008) indicates that the body of this volume lies within three major tracts: inferior fronto-occipital fasciculus, inferior longitudinal fasciculus, and the forceps major. For comparison with previous work, the tract between LGN and hMT+/V5 derived from the healthy hemisphere of nine participants in a previous study has been superimposed (Willis et al., 2024a). While the VLSM cluster is not in the center of this independently derived tract, there is some overlap.

## Discussion

In the current study, we investigated the relationship between structural damage to the occipital cortex and residual vision in people with damage to V1 resulting in homonymous visual field loss. We aimed to determine the areas of the occipital cortex essential for residual vision. Although we hypothesized that the extent of damage to dorsal and ventral areas, hMT+/V5 and V4, would correlate with performance on motion and face/place tasks, respectively, it was damage to hMT+/V5 that resulted in poor residual vision across all tasks.

The use of correlations to distinguish between structural damage and visual task performance was complicated by the difference in the number of participants across the tasks. Thus, much of the discussion is about the major patterns in the data rather than differences in significance levels. The effect of *n* is particularly evident across the three tasks for area hMT+/V5 where the correlation levels are comparable, yet only the contrast task was statistically significant. The confidence intervals for each correlation were presented to reduce the reliance on the significance level.

### The importance of hMT+/V5

Processing of motion stimuli after damage to V1 specifically activates extrastriate hMT+/V5, suggesting that existing pathways that bypass V1 might underlie this residual vision. While the number of patients in the current study with damage to hMT+/V5 was relatively small (8/39), none showed residual vision for either the motion stimulus or for face/place stimuli. Only two participants with small amounts of damage (2 and 20%) performed well on the contrast tasks, with all others at chance, indicating a critical role for this area. As this atlas-defined region exhibits relatively high interindividual variability, there is a chance that smaller lesions could fall outside its true location. Also, hMT+/V5 here likely encompasses MT and MST, implying that either subregion would be critical to detect moving dots. One possible explanation for the profound impact of lesions in many cases is that they were defined manually using T1 scans, which may underestimate lesion involvement, e.g., compared with quantitative MRI (Seiler et al., 2021). Although this would not impact regression analyses due to affecting all regions equally, it is relevant when considering binary loss of function.

Recent work has shown an asymmetry in hMT+/V5 processing in humans, such that the right hemisphere preferentially processes motion integration (Chiappini et al., 2022; Pitcher, 2022) and seems to integrate across both hemifields (Strong et al., 2019). This raises the question of whether there are differences in visual impairment depending on whether the damage is to the left or right hemisphere, particularly when it extends to hMT+/V5. Only eight of the participants in the current study showed damage that extended into hMT+/V5, and there was no obvious difference in visual performance on the tasks between those with damage to the right (*n* = 5) or left (*n* = 3) hemisphere. However, this would be an interesting question to address in more detail in future studies.

Conversely, we also found that some participants without damage to hMT+/V5 still show poor residual vision. This suggests that damage to hMT+/V5 alone cannot explain whether an individual is likely to show residual vision. This is where the VLSM analysis provided additional insight, as it was damage to the optic radiation and lateral visual pathways that were implicated in poor residual vision. This is consistent with the previous finding that the white matter pathway between LGN and hMT+/V5 was compromised in patients who did not show blindsight but intact in those who did (Ajina et al., 2015b).

### Damage to ventral area V4 does not affect the detection of faces and places

Perhaps the most surprising finding was that the amount of damage to V4 did not significantly correlate with the detection of faces and places. V4 is known to respond to several surface properties including color, luminance, texture, and shape (Roe et al., 2012) and has been shown to be active during presentation of static, colored images in the blind field (Goebel et al., 2001). It therefore may be expected to be involved in the detection of stimuli with these components. In contrast, there was a significant correlation between the ability to detect the contrast stimulus and the extent of damage to V4. Notably, there were some participants with extensive damage to V4 who were able to perform all tasks. In a previous study using VLSM, Spang et al. (2021) investigated the cortical areas implicated in the impairment of luminance, texture, motion, and color detection and discrimination in stroke survivors with occipital, temporal, or parietal damage, but spared central vision. The patient population differed slightly from the current study as posterior V1 is unlikely to be affected, and therefore the patients did not have a visual field deficit. However, despite the absence of a visual field deficit, several of the patients exhibited impaired visual performance in the visual hemifield contralateral to the damage. Their VLSM analysis indicated that impairment in visual performance tasks was associated with damage in the region of V4. We therefore expected to find a similar effect of damage to V4 on face and place detection. However, Spang et al. (2021) found that detection thresholds were only weakly correlated when compared with discrimination thresholds. The difference may therefore relate to the use of detection tasks in the current study.

While there is little doubt that V4 is involved in processing form, including concentric and radial form and faces (Wilkinson et al., 2000), identification of the face or places was not necessary for the tasks used here. To better understand the role of V4 in residual form vision, it will be necessary to perform a more detailed investigation of form, texture, color, and object perception across different types of tasks in homonymous visual field deficits. It would also be useful to understand its dependence on V5/hMT+ in static image detection, as both the current study and previous work (Goebel et al., 2001) suggest V5/hMT+ may also be involved in the processing of static, colored images in blindsight.

### Identifying regions critical for residual vision following V1 damage

The current study used structural data to investigate the key structures underlying residual vision in a relatively large study population. A major finding was that the white matter adjacent to V5/hMT+ was necessary for residual vision. This is broadly consistent with a previous study that used diffusion-weighted imaging and indicated the requirement for an intact tract between LGN and V5/hMT+ to demonstrate residual vision (Ajina et al., 2015b). Similarly, functional MRI connectivity has shown a strong correlation between the BOLD signal in V5/hMT+ and LGN in those with residual vision (Ajina and Bridge, 2018). Thus three different imaging modalities suggest the importance of this connection, although the damage could be to the white matter or the gray matter of V5/hMT+. A future critical approach could be to analyze all of these MRI metrics together to determine whether they are consistent.

### Limitations of the study

Although three different stimulus types were used, they all used the same experimental protocol and did not require stimulus identification or discrimination. They did, however, differ according to the extent of salience which could affect the performance of participants. For example, the motion stimulus consisted of black dots on a midgray background, whereas the face/place stimuli contained some color information. The double dissociation for detection and discrimination tasks is evident in the large study of Spang et al. (2021), suggesting different task demands could also lead to different results.

The study only considered a single visual location for the behavioral testing which was located fully within the Humphrey perimetry–defined blind field in each case. In many cases, the blind region would be significantly larger than the stimulus size, and therefore there are regions that are untested. Since the regions included in the lesion are not limited to the tested area, it may be that a different location could have yielded a different outcome. However, this could only be determined using extensive behavioral testing.

Furthermore, the study only used a limited number of visual areas, principally because it is not straightforward to individually map visual areas in patients with hemianopia using retinotopic mapping (Papanikolaou et al., 2014; Halbertsma et al., 2021). While there are retinotopic atlases that can be used (Wang et al., 2015; Benson and Winawer, 2018), the accuracy of such approaches in brains with lesions is not well understood.

Finally, one of the interesting aspects of residual vision is the extent to which people report some form of awareness for stimuli projected in their clinically blind field (such as type II blindsight and the Riddoch phenomenon) or are entirely unaware of its presence (type I blindsight; Danckert et al., 2019). Awareness measures were not systematically taken across all participants meaning that it was not possible to determine whether awareness varies according to the specific location of the occipital damage.

### Considerations for rehabilitation training

The results of the current study may be able to inform rehabilitation training. In recent years, a large number of studies have found that training within the blind field can improve vision in those with damage to V1 (Huxlin, 2008; Huxlin et al., 2009; Elshout et al., 2016; Cavanaugh and Huxlin, 2017; Cavanaugh et al., 2019; Ajina et al., 2021; Willis et al., 2024a). All these studies, however, have shown that visual rehabilitation of hemianopia requires a large time commitment involving many months of training, and does not benefit everyone equally. Thus far, there is little evidence as to why some patients benefit from these extensive visual rehabilitation and others do not. Two small imaging studies have shown that baseline BOLD activity in V5/hMT+ (Ajina et al., 2021) and perilesional V1 (Barbot et al., 2021) predict rehabilitation success. Moreover, rehabilitation improved the white matter microstructure in the tract between hMT+/V5 and LGN (Willis et al., 2024a). In the current study, we found that damage to hMT+/V5 or the optic radiation significantly affects residual visual abilities. It is possible that limited residual vision at baseline due to damage to hMT+/V5 or optic tract may also link to poor rehabilitation outcomes.

A further point for consideration is that the majority of visual rehabilitation methods use moving stimuli, which may be ineffective if the individual has damage to hMT+/V5. It is therefore possible that lesion size or location before training may be able to inform the most successful method. For example, if a participant has damage to hMT+/V5, but intact V4, it may be optimal to provide rehabilitation that trains the ventral, rather than dorsal visual pathway.

## Conclusion

Investigating residual vision in a uniquely large group of people with hemianopia and visual field loss has allowed us to apply the method of voxel-based lesion–symptom mapping (VLSM) to measure the direct relationship between lesion location and residual visual function. This indicated that beyond hMT+/V5 being important for residual vision, the preservation of the lateral optic radiation is most highly associated with residual vision.
